# Supplementary material for: The Cytomegalovirus M35 Protein Directly Binds to the Interferon-β Enhancer and Modulates Transcription of Ifnb1 and Other IRF3-Driven Genes
Source: J Virol. 2023 Jun 8;97(6):e00400-23. doi: 10.1128/jvi.00400-23 (PMC10308904; doi:10.1128/jvi.00400-23)
Supplement: Supplemental file 1 — File S1. Download jvi.00400-23-s0002.pdf, PDF file, 0.4 MB [file jvi.00400-23-s0002.pdf]

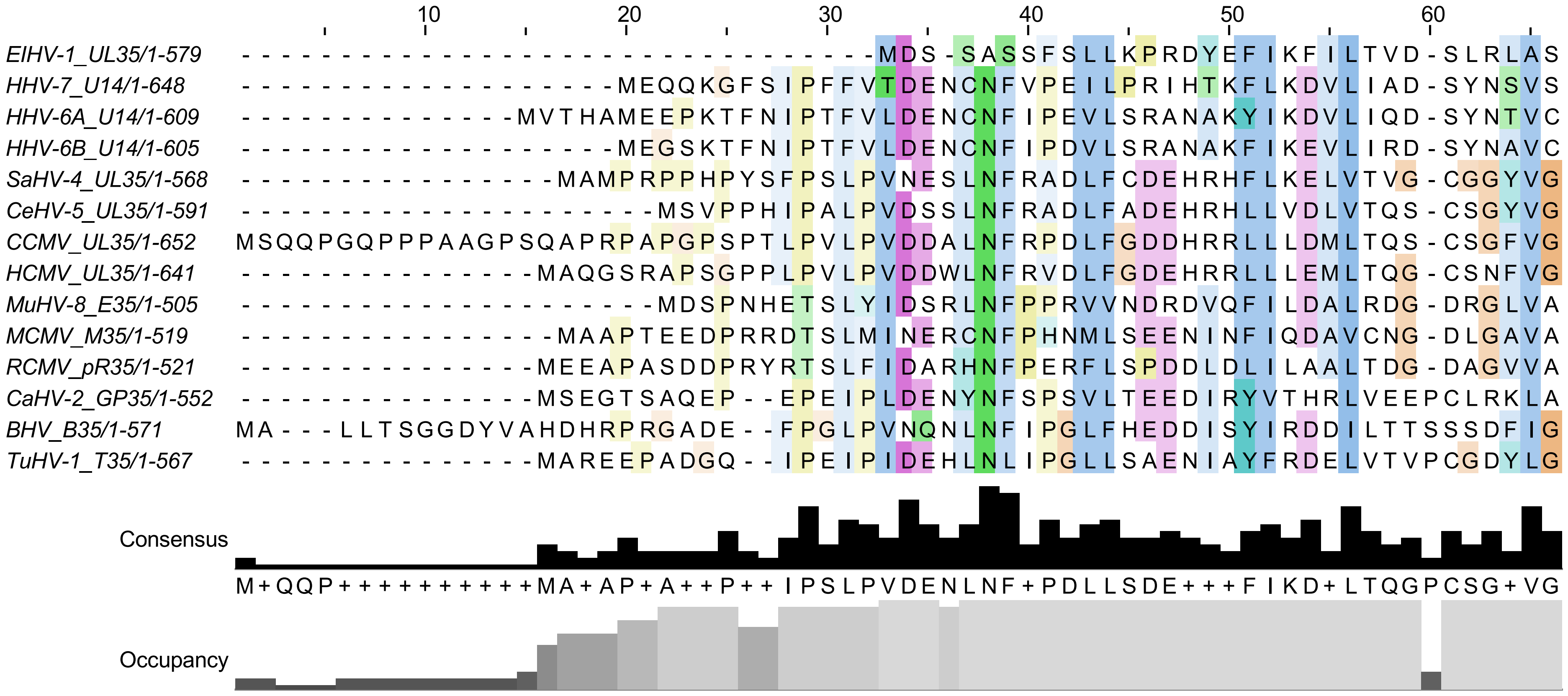
10 20 30 40 50 60
EIHV-1_UL35/1-579 --------------------------------MDS-SASSFSLLKPRDYEFIKFILTVD-SLRLAS
HHV-7_U14/1-648 --------------------MEQQKGFSIPFFVTDENCNFVPEILPRIHTKFLKDVLIAD-SYNSVS
HHV-6A_U14/1-609 -----------------MVTHAMEEPKTFNIPTFVLDENCNFIPEVLSRANAKYIKDVLIQD-SYNTVC
HHV-6B_U14/1-605 ---------------------MEGSKTFNIPTFVLDENCNFIPDVLSRANAKFIKEVLIRD-SYNAVC
SaHV-4_UL35/1-568 -----------------MAMPRPPHPYSFPSLPVNESLNFRADLFCDEHRHFLKELVTVG-CGGYVG
CeHV-5_UL35/1-591 --------------------MSVPPHIPALPVDSSLNFRADLFADEHRHLLVDLVTQS-CSGYVG
CCMV_UL35/1-652 MSQQPGQPPPAAGPSQAPRPAPGPSPTLPVLPVDDALNFRPDLFGDDHRRLLLDMLTQS-CSGFVG
HCMV_UL35/1-641 ----------------MAQGSRAPSGPPLPVLPVDDWLNFRVDLFGDEHRRLLLEMLTQG-CSNFVG
MuHV-8_E35/1-505 --------------------MDSPNHETSLYIDSRLNFPPRVVNDRDVQFILDALRDG-DRGLVA
MCMV_M35/1-519 -----------------MAAPTEEDPRRDTSLMINERCNFPHNMLSEENINFIQDAVCNG-DLGAVA
RCMV_pR35/1-521 ----------------MEEAPASDDPRYRTSLFIDARHNFPERFLSPDDLDLILAALTDG-DAGVVA
CaHV-2_GP35/1-552 ----------------MSEGTSAQEP--EPEIPLDENYNFSPSVLTEEDIRYVTHRLVEEPCLRKLA
BHV_B35/1-571 MA---LLTSGGDYVAHDHRPRGADE--FPGLPVNQNLNFIPGLFHEDDISYIRDDILTTSSSDFIG
TuHV-1_T35/1-567 ----------------MAREEPADGQ--IPEIPIDEHLNLIPGLLSAENIAYFRDELVTVPCGDYLG
Consensus
M+QQP+++++++++MA+AP+A++P++IPSLPVDENLNF+PDLLSDE+++FIKD+LTQGPCSG+VG
Occupancy

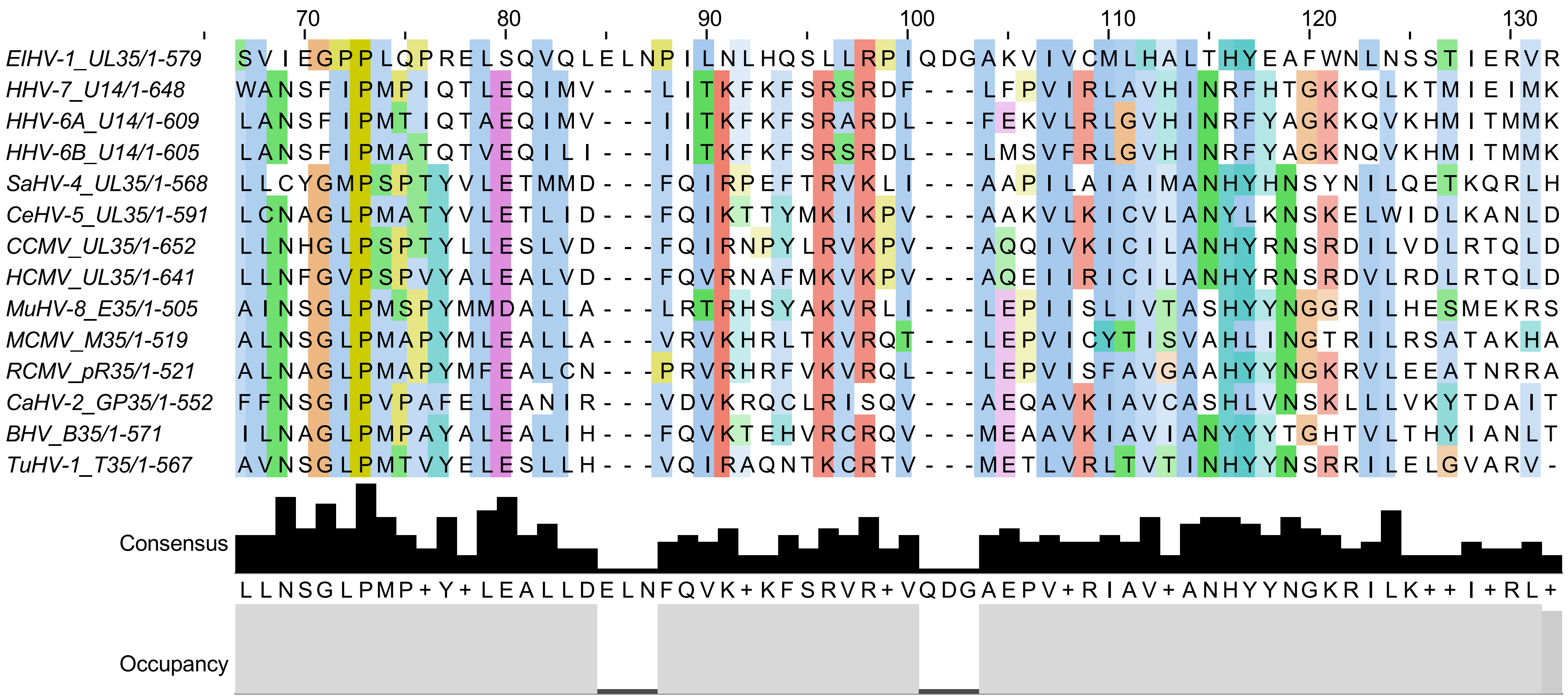
70 80 90 100 110 120 130
EIHV-1_UL35/1-579 SVIEGPPLQPRELSQVQLELNPILNLHQSLLRPIQDGAKVIVCMLHALTHYEAFWNLNSSTIERVR
HHV-7_U14/1-648 WANSFIPMPIQTLEQIMV---LITKFKFSRSRDF---LFPVIRLAVHINRFHTGKKQLKTMIEIMK
HHV-6A_U14/1-609 LANSFIPMTIQTAEQIMV---IITKFKFSRARDL---FEKVLRLGVHINRFYAGKKQVKHMITMMK
HHV-6B_U14/1-605 LANSFIPMATQTVEQILI---IITKFKFSRSRDL---LMSVFRLGVHINRFYAGKNQVKHMITMMK
SaHV-4_UL35/1-568 LLCYGMPSPTYVLETMMD---FQIRPEFTRVKLI---AAPILAIAIMANHYHNSYNILQETKQRLH
CeHV-5_UL35/1-591 LCNAGLPMATYVLETLID---FQIKTTYMKIKPV---AAKVLKICVLANYLKNSKELWIDLKANLD
CCMV_UL35/1-652 LLNHGLPSPTYLLESLVD---FQIRNPYLRVKPV---AQQIVKICILANHYRNSRDILVDLRTQLD
HCMV_UL35/1-641 LLNFGVPSPVYALEALVD---FQVRNAFMKVKPV---AQEIIRICILANHYRNSRDVLRDLRTQLD
MuHV-8_E35/1-505 AINSGLPMSPYMMDALLA---LRTRHSYAKVRLI---LEPIISLIVTASHYYNGGRILHESMEKRS
MCMV_M35/1-519 ALNSGLPMAPYMLEALLA---VRVKHRLTKVRQT---LEPVICYTISVAHLINGTRILRSATAKHA
RCMV_pR35/1-521 ALNAGLPMAPYMFEALCN---PRVRHRFVKVRQL---LEPVISFAVGAAHYYNGKRVLEEATNRRA
CaHV-2_GP35/1-552 FFNSGIPVPAFELEANIR---VDVKRQCLRISQV---AEQAVKIAVCASHLVNSKLLLVKYTDAIT
BHV_B35/1-571 ILNAGLPMPAYALEALIH---FQVKTEHVRCRQV---MEAAVKIAVIANYYYTGHTVLTHYIANLT
TuHV-1_T35/1-567 AVNSGLPMTVYELESLLH---VQIRAQNTKCRTV---METLVRLTVTINHYYNSRRILELGVARV-
Consensus
LLNSGLPMP+Y+LEALLDELNFQVK+KFSRVR+VQDGAEPV+RIAV+ANHYYNGKRILK++I+RL+
Occupancy

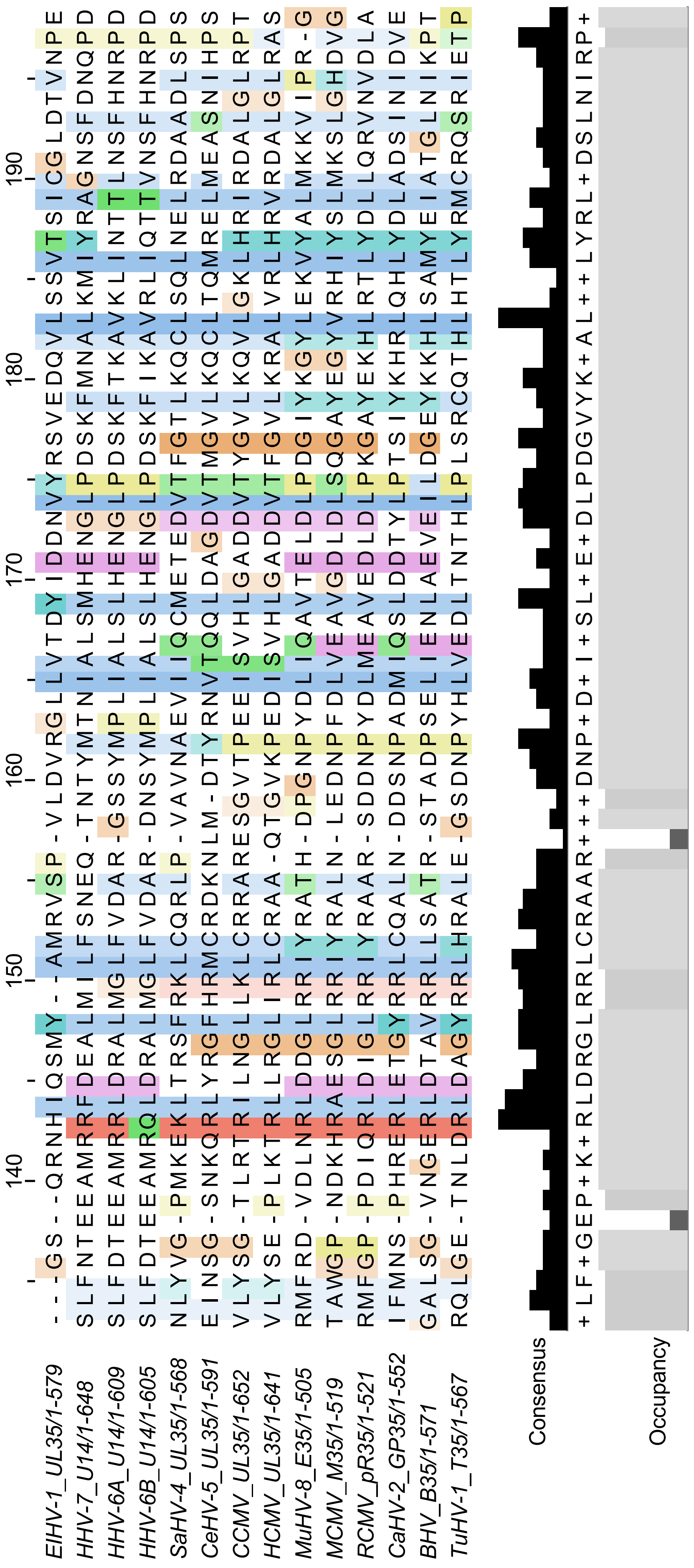

EIHV-1_UL35/1-579
HHV-7_U14/1-648
HHV-6A_U14/1-609
HHV-6B_U14/1-605
SaHV-4_UL35/1-568
CeHV-5_UL35/1-591
CCMV_UL35/1-652
HCMV_UL35/1-641
MuHV-8_E35/1-505
MCMV_M35/1-519
RCMV_pR35/1-521
CaHV-2_GP35/1-552
BHV_B35/1-571
TuHV-1_T35/1-567
Consensus
Occupancy
140
150
160
170
180
190

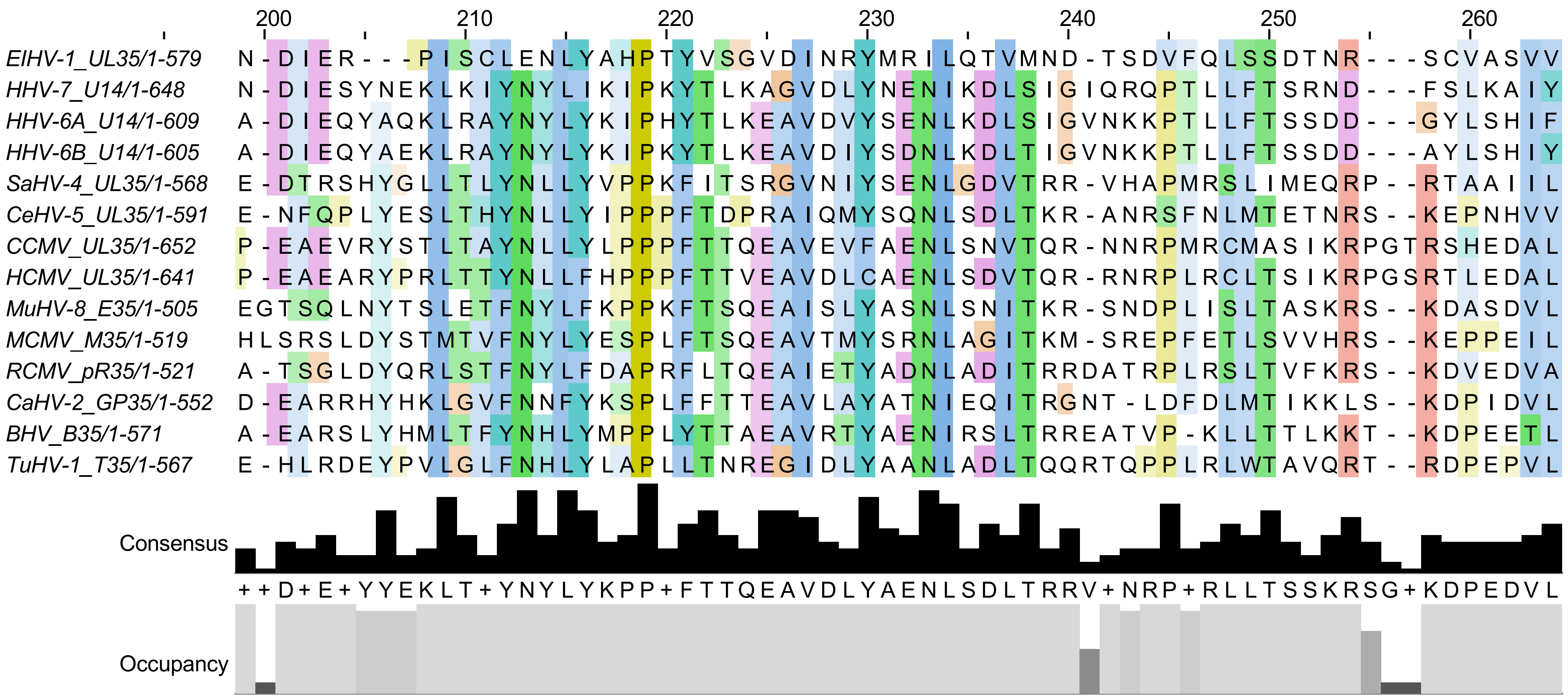

EIHV-1_UL35/1-579
HHV-7_U14/1-648
HHV-6A_U14/1-609
HHV-6B_U14/1-605
SaHV-4_UL35/1-568
CeHV-5_UL35/1-591
CCMV_UL35/1-652
HCMV_UL35/1-641
MuHV-8_E35/1-505
MCMV_M35/1-519
RCMV_pR35/1-521
CaHV-2_GP35/1-552
BHV_B35/1-571
TuHV-1_T35/1-567
Consensus
Occupancy
200
210
220
230
240
250
260

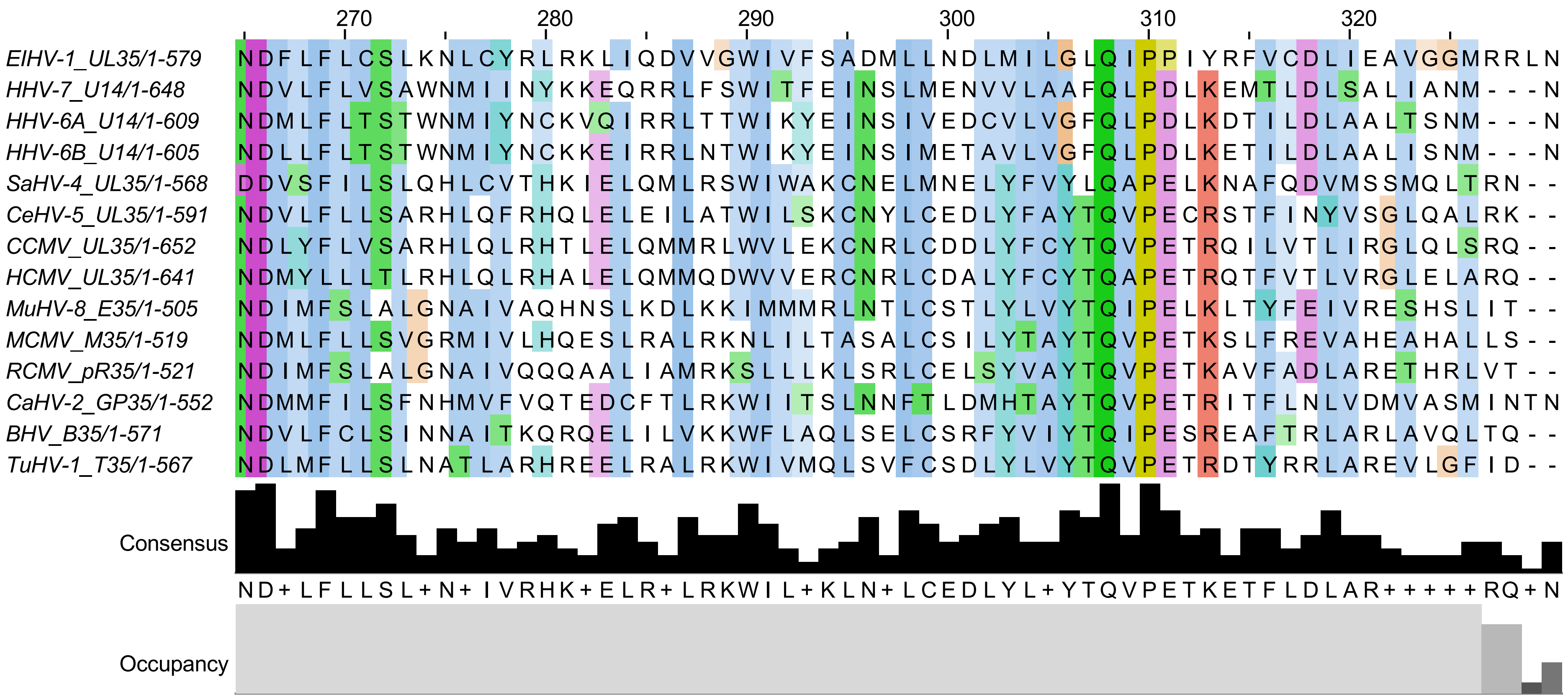
270 280 290 300 310 320
EIHV-1_UL35/1-579 NDFLFLCSLKNLCYRLRKLIQDVVGWIVFSADMLLNDLMILGLQIPPIYRFVCDLIEAVGGMRRLN
HHV-7_U14/1-648 NDVLFLVSAWNMIINYKKEQRRLFSWITFEINSLMENVVLAAFQLPDLKEMTLDLSALIANM---N
HHV-6A_U14/1-609 NDMLFLTSTWNMIYNCKVQIRRLTTWIKYEINSIVEDCVLVGFQLPDLKDTILDLAALTSNM---N
HHV-6B_U14/1-605 NDLLFLTSTWNMIYNCKKEIRRLNTWIKYEINSIMETAVLVGFQLPDLKETILDLAALISNM---N
SaHV-4_UL35/1-568 DDVSFILSLQHLCVTHKIELQMLRSWIWAKCNELMNELYFVYLQAPELKNAFQDVMSSMQLTRN--
CeHV-5_UL35/1-591 NDVLFLLSARHLQFRHQLELEILATWILSKCNYLCEDLYFAYTQVPECRSTFINYVSGLQALRK--
CCMV_UL35/1-652 NDLYFLVSARHLQLRHTLELQMMRLWVLEKCNRLCDDLYFCYTQVPETRQILVTLIRGLQLSRQ--
HCMV_UL35/1-641 NDMYLLLTLRHLQLRHALELQMMQDWVVERCNRLCDALYFCYTQAPETRQTFVTLVRGLELARQ--
MuHV-8_E35/1-505 NDIMFSLALGNAIVAQHNSLKDLKKIMMMRLNTLCSTLYLVYTQIPELKLTYFEIVRESHSLIT--
MCMV_M35/1-519 NDMLFLLSVGRMIVLHQESLRALRKNLILTASALCSILYTAYTQVPETKSLFREVAHEAHALLS--
RCMV_pR35/1-521 NDIMFSLALGNAIVQQQAALIAMRKSLLLKLSRLCELSYVAYTQVPETKAVFADLARETHRLVT--
CaHV-2_GP35/1-552 NDMMFILSFNHMVFVQTEDCFTLRKWIITSLNNFTLDMHTAYTQVPETRITFLNLVDMVASMINTN
BHV_B35/1-571 NDVLFCLSINNAITKQRQELILVKKWFLAQLSELCSRFYVIYTQIPESREAFTRLARLAVQLTQ--
TuHV-1_T35/1-567 NDLMFLLSLNATLARHREELRALRKWIVMQLSVFCSDLYLVYTQVPETRDTYRRLAREVLGFID--
Consensus
ND+LFLLSL+N+IVRHK+ELR+LRKWIL+KLN+LCEDLYL+YTQVPETKETFLDLAR+++++RQ+N
Occupancy

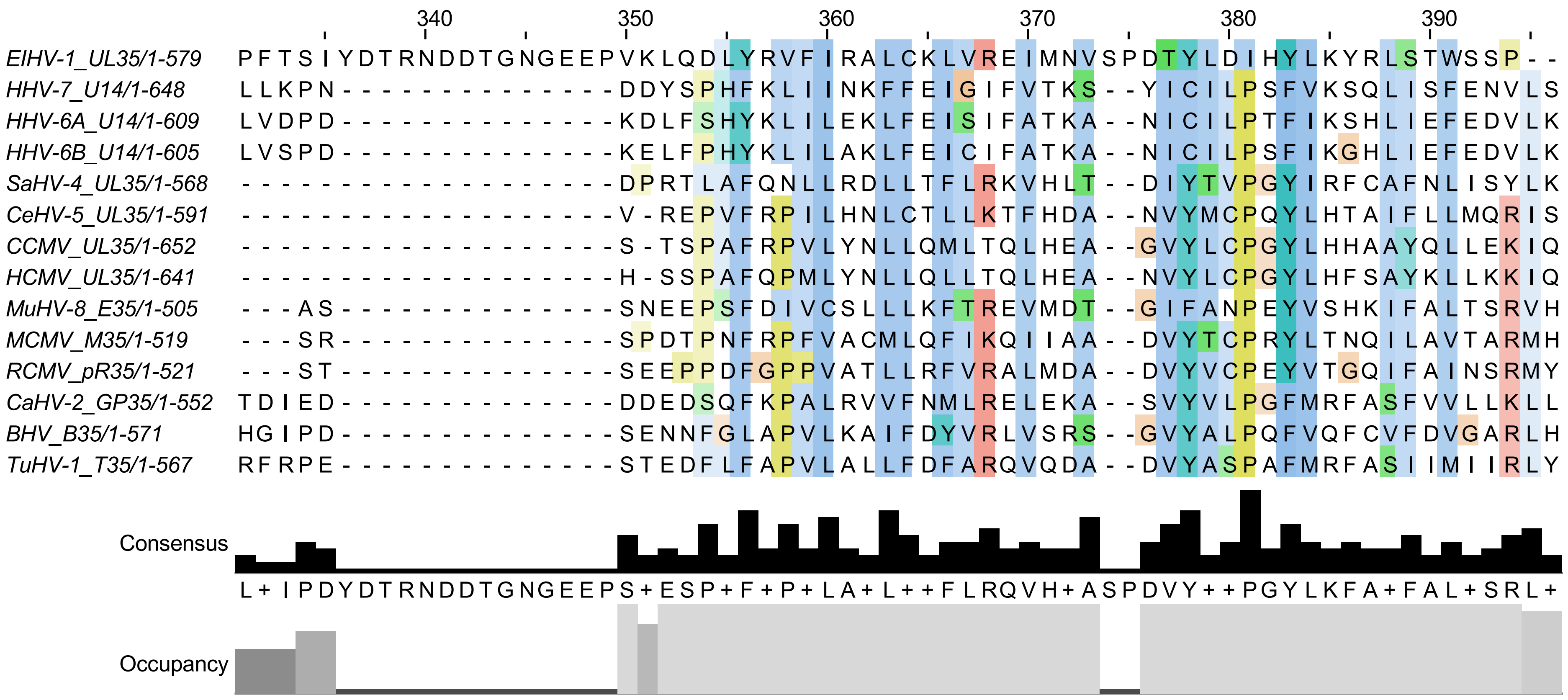

340 350 360 370 380 390
EIHV-1_UL35/1-579 PFTSIYDTRNDDTGNGEEPVKLQDLYRVFIRALCKLVREIMNVSPDTYLDIHYLKYRLSTWSSP--
HHV-7_U14/1-648 LLKPN--------------DDYSPHFKLIINKFFEIGIFVTKS--YICILPSFVKSQLISFENVLS
HHV-6A_U14/1-609 LVDPD--------------KDLFSHYKLILEKLFEISIFATKA--NICILPTFIKSHLIEFEDVLK
HHV-6B_U14/1-605 LVSPD--------------KELFPHYKLILAKLFEICIFATKA--NICILPSFIKGHLIEFEDVLK
SaHV-4_UL35/1-568 -------------------DPRTLAFQNLLRDLLTFLRKVHLT--DIYTVPGYIRFCAFNLISYLK
CeHV-5_UL35/1-591 -------------------V-REPVFRPILHNLCTLLKTFHDA--NVYMCPQYLHTAIFLLMQRIS
CCMV_UL35/1-652 -------------------S-TSPAFRPVLYNLLQMLTQLHEA--GVYLCPGYLHHAAYQLLEKIQ
HCMV_UL35/1-641 -------------------H-SSPAFQPMLYNLLQLLTQLHEA--NVYLCPGYLHFSAYKLLKKIQ
MuHV-8_E35/1-505 ---AS--------------SNEEPSFDIVCSLLLKFTREVMDT--GIFANPEYVSHKIFALTSRVH
MCMV_M35/1-519 ---SR--------------SPDTPNFRPFVACMLQFIKQIIAA--DVYTCPRYLTNQILAVTARMH
RCMV_pR35/1-521 ---ST--------------SEEPPDFGPPVATLLRFVRALMDA--DVYVCPEYVTGQIFAINSRMY
CaHV-2_GP35/1-552 TDIED--------------DDEDSQFKPALRVVFNMLRELEKA--SVYVLPGFMRFASFVVLLKLL
BHV_B35/1-571 HGIPD--------------SENNFGLAPVLKAIFDYVRLVSRS--GVYALPQFVQFCVFDVGARLH
TuHV-1_T35/1-567 RFRPE--------------STEDFLFAPVLALLFDFARQVQDA--DVYASPAFMRFASIIMIIRLY
Consensus
L+IPDYDTRNDDTGNGEEPS+ESP+F+P+LA+L++FLRQVH+ASPDVY++PGYLKFA+FAL+SRL+
Occupancy

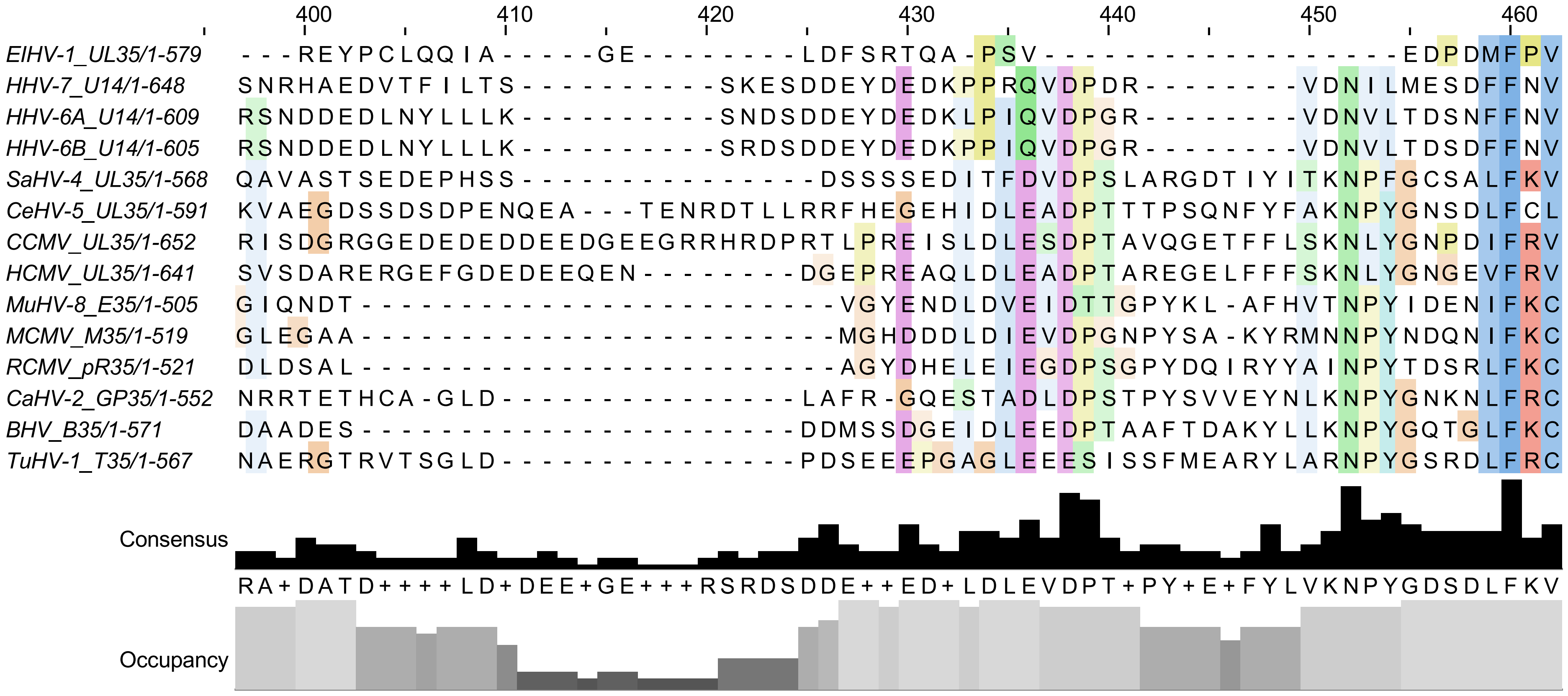
400
410
420
430
440
450
460
EIHV-1_UL35/1-579 ---REYPCLQQIA-----GE--------LDFSRTQA-PSV------------------EDPDMFPV
HHV-7_U14/1-648 SNRHAEDVTFILTS----------SKESDDEYDEDKPPRQVDPDR--------VDNILMESDFFNV
HHV-6A_U14/1-609 RSNDDEDLNYLLLK----------SNDSDDEYDEDKLPIQVDPGR--------VDNVLTDSNFFNV
HHV-6B_U14/1-605 RSNDDEDLNYLLLK----------SRDSDDEYDEDKPPIQVDPGR--------VDNVLTDSDFFNV
SaHV-4_UL35/1-568 QAVASTSEDEPHSS---------------DSSSSEDITFDVDPSLARGDTIYITKNPFGCSALFKV
CeHV-5_UL35/1-591 KVAEGDSSDSDPENQEA---TENRDTLLRRFHEGEHIDLEADPTTTPSQNFYFAKNPYGNSDLFCL
CCMV_UL35/1-652 RISDGRGGEDEDEDDEEDGEEGRRHRDPRTLPREISLDLESDPTAVQGETFFLSKNLYGNPDIFRV
HCMV_UL35/1-641 SVSDARERGEFGDEDEEQEN--------DGEPREAQLDLEADPTAREGELFFFSKNLYGNGEVFRV
MuHV-8_E35/1-505 GIQNDT------------------------VGYENDLDVEIDTTGPYKL-AFHVTNPYIDENIFKC
MCMV_M35/1-519 GLEGAA------------------------MGHDDDLDIEVDPGNPYSA-KYRMNNPYNDQNIFKC
RCMV_pR35/1-521 DLDSAL------------------------AGYDHELEIEGDPSGPYDQIRYYAINPYTDSRLFKC
CaHV-2_GP35/1-552 NRRTETHCA-GLD---------------LAFR-GQESTADLDPSTPYSVVEYNLKNPYGNKNLFRC
BHV_B35/1-571 DAADES----------------------DDMSSDGEIDLEEDPTAAFTDAKYLLKNPYGQTGLFKC
TuHV-1_T35/1-567 NAERGTRVTSGLD---------------PDSEEEPGAGLEEESISSFMEARYLARNPYGSRDLFRC
Consensus
RA+DATD++++LD+DEE+GE+++RSRDSDDE++ED+LDLEVDPT+PY+E+FYLVKNPYGDSDLFKV
Occupancy

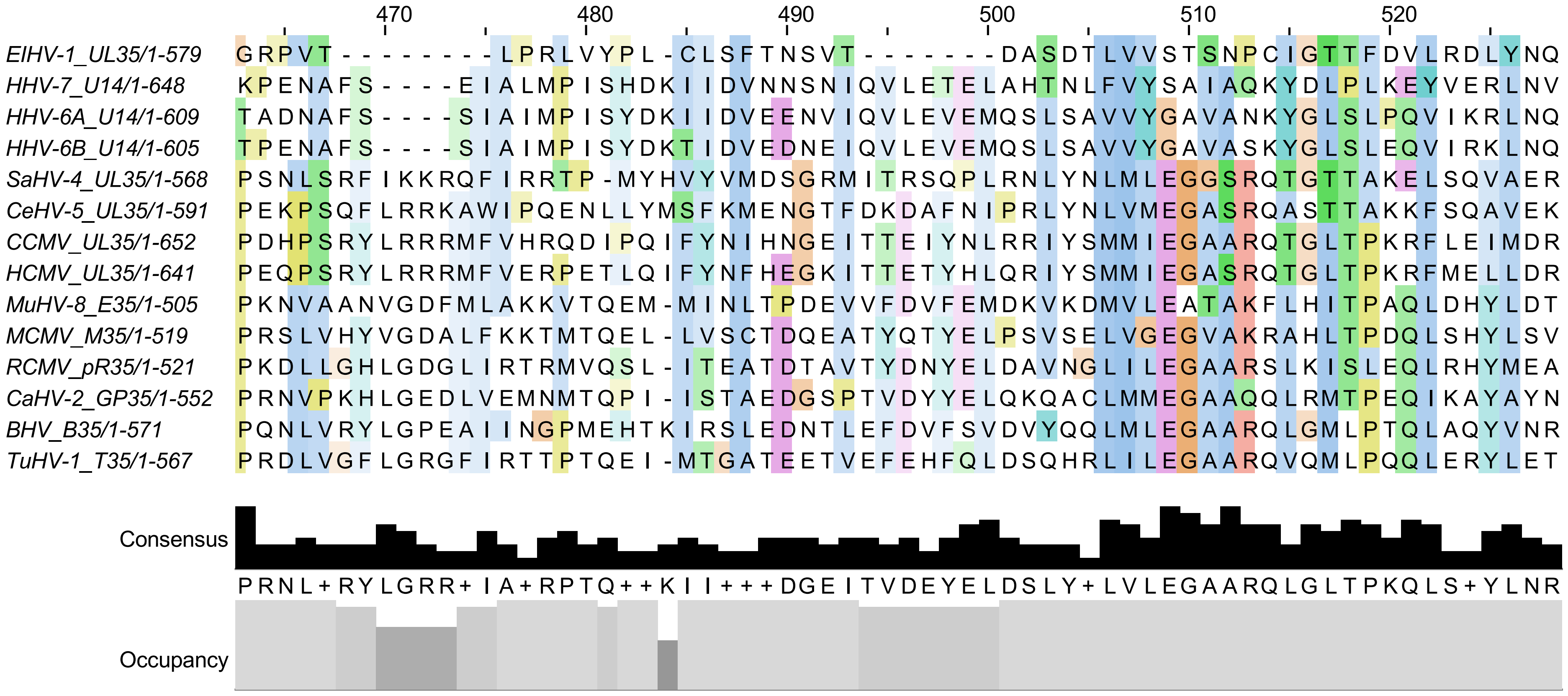

470
480
490
500
510
520
EIHV-1_UL35/1-579 GRPVT--------LPRLVYPL-CLSFTNSVT-------DASDTLVVSTSNPCIGTTFDVLRDLYNQ
HHV-7_U14/1-648 KPENAFS----EIALMPISHDKIIDVNNSNIQVLETELAHTNLFVYSAIAQKYDLPLKEYVERLNV
HHV-6A_U14/1-609 TADNAFS----SIAIMPISYDKIIDVEENVIQVLEVEMQSLSAVVYGAVANKYGLSLPQVIKRLNQ
HHV-6B_U14/1-605 TPENAFS----SIAIMPISYDKTIDVEDNEIQVLEVEMQSLSAVVYGAVASKYGLSLEQVIRKLNQ
SaHV-4_UL35/1-568 PSNLSRFIKKRQFIRRTP-MYHVYVMDSGRMITRSQPLRNLYNLMLEGGSRQTGTTAKELSQVAER
CeHV-5_UL35/1-591 PEKPSQFLRRKAWIPQENLLYMSFKMENGTFDKDAFNIPRLYNLVMEGASRQASTTAKKFSQAVEK
CCMV_UL35/1-652 PDHPSRYLRRRMFVHRQDIPQIFYNIHNGEITTEIYNLRRIYSMMIEGAARQTGLTPKRFLEIMDR
HCMV_UL35/1-641 PEQPSRYLRRRMFVERPETLQIFYNFHEGKITTETYHLQRIYSMMIEGASRQTGLTPKRFMELLDR
MuHV-8_E35/1-505 PKNVAANVGDFMLAKKVTQEM-MINLTPDEVVFDVFEMDKVKDMVLEATAKFLHITPAQLDHYLDT
MCMV_M35/1-519 PRSLVHYVGDALFKKTMTQEL-LVSCTDQEATYQTYELPSVSELVGEGVAKRAHLTPDQLSHYLSV
RCMV_pR35/1-521 PKDLLGHLGDGLIRTRMVQSL-ITEATDTAVTYDNYELDAVNGLILEGAARSLKISLEQLRHYMEA
CaHV-2_GP35/1-552 PRNVPKHLGEDLVEMNMTQPI-ISTAEDGSPTVDYYELQKQACLMMEGAAQQLRMTPEQIKAYAYN
BHV_B35/1-571 PQNLVRYLGPEAIINGPMEHTKIRSLEDNTLEFDVFSVDVYQQLMLEGAARQLGMLPTQLAQYVNR
TuHV-1_T35/1-567 PRDLVGFLGRGFIRTTPTQEI-MTGATEETVEFEHFQLDSQHRLILEGAARQVQMLPQQLERYLET
Consensus
PRNL+RYLGRR+IA+RPTQ++KII+++DGEITVDEYELDSLY+LVLEGAARQLGLTPKQLS+YLNR
Occupancy

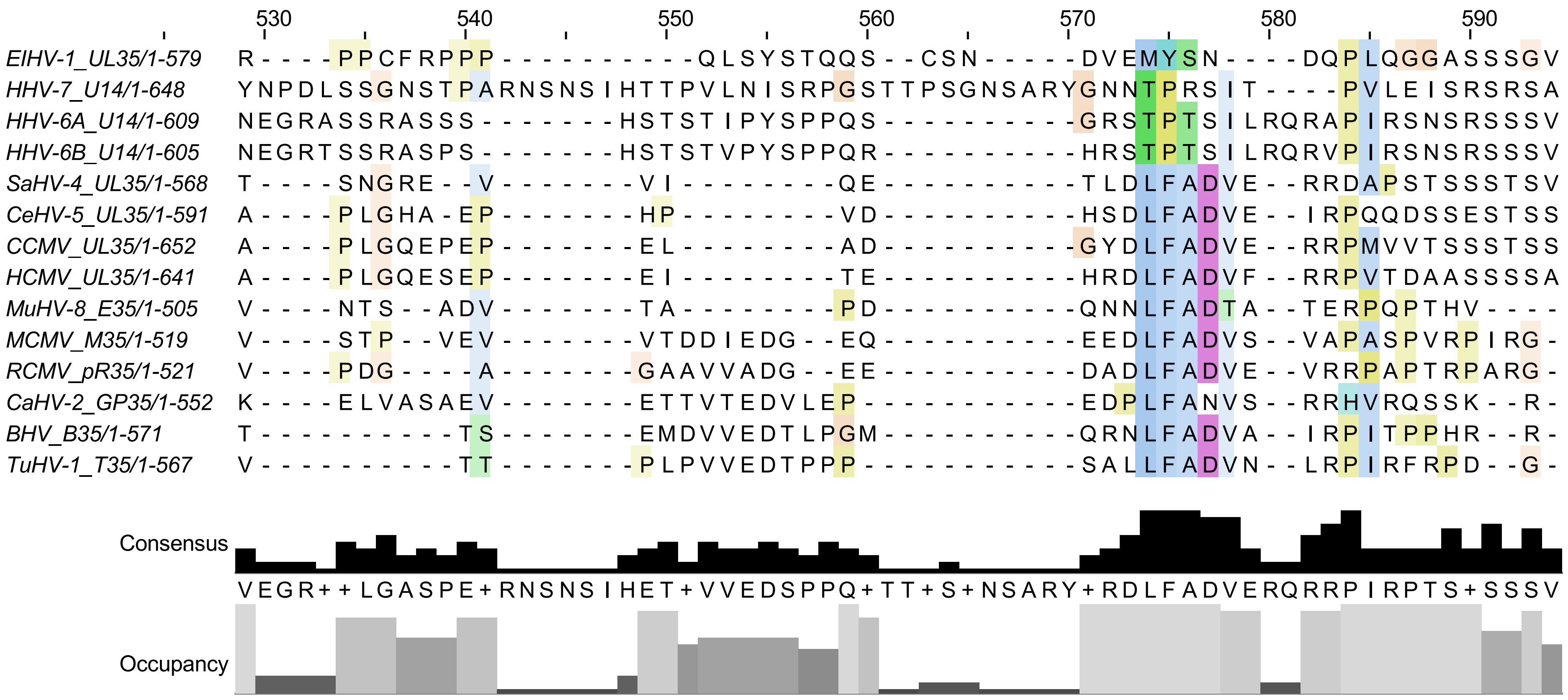
530 540 550 560 570 580 590
ElHV-1_UL35/1-579 R - - - - P P C F R P P P - - - - - - - - - - - Q L S Y S T Q Q S - - C S N - - - - - D V E M Y S N - - - - D Q P L Q G G A S S S G V
HHV-7_U14/1-648 Y N P D L S S G N S T P A R N S N S I H T T P V L N I S R P G S T T P S G N S A R Y G N N T P R S I T - - - - P V L E I S R S R S A
HHV-6A_U14/1-609 N E G R A S S R A S S S - - - - - - - H S T S T I P Y S P P Q S - - - - - - - - - - G R S T P T S I L R Q R A P I R S N S R S S S V
HHV-6B_U14/1-605 N E G R T S S R A S P S - - - - - - - H S T S T V P Y S P P Q R - - - - - - - - - - H R S T P T S I L R Q R V P I R S N S R S S S V
SaHV-4_UL35/1-568 T - - - - S N G R E - - V - - - - - - - V I - - - - - - - - - Q E - - - - - - - - - - T L D L F A D V E - - R R D A P S T S S S T S V
CeHV-5_UL35/1-591 A - - - - P L G H A - E P - - - - - - - H P - - - - - - - - - V D - - - - - - - - - - H S D L F A D V E - - I R P Q Q D S S E S T S S
CCMV_UL35/1-652 A - - - - P L G Q E P E P - - - - - - - E L - - - - - - - - - A D - - - - - - - - - - G Y D L F A D V E - - R R P M V V T S S S T S S
HCMV_UL35/1-641 A - - - - P L G Q E S E P - - - - - - - E I - - - - - - - - - T E - - - - - - - - - - H R D L F A D V F - - R R P V T D A A S S S S A
MuHV-8_E35/1-505 V - - - - N T S - - A D V - - - - - - - T A - - - - - - - - - P D - - - - - - - - - - Q N N L F A D T A - - T E R P Q P T H V - - - -
MCMV_M35/1-519 V - - - - S T P - - V E V - - - - - - - V T D D I E D G - - E Q - - - - - - - - - - E E D L F A D V S - - V A P A S P V R P I R G -
RCMV_pR35/1-521 V - - - - P D G - - - - A - - - - - - - G A A V V A D G - - E E - - - - - - - - - - D A D L F A D V E - - V R R P A P T R P A R G -
CaHV-2_GP35/1-552 K - - - - E L V A S A E V - - - - - - - E T T V T E D V L E P - - - - - - - - - - - E D P L F A N V S - - R R H V R Q S S K - - R -
BHV_B35/1-571 T - - - - - - - - - - T S - - - - - - - E M D V V E D T L P G M - - - - - - - - - - Q R N L F A D V A - - I R P I T P P H R - - R -
TuHV-1_T35/1-567 V - - - - - - - - - - T T - - - - - - - P L P V V E D T P P P - - - - - - - - - - - S A L L F A D V N - - L R P I R F R P D - - G -
Consensus
V E G R + + L G A S P E + R N S N S I H E T + V V E D S P P Q + T T + S + N S A R Y + R D L F A D V E R Q R R P I R P T S + S S S V
Occupancy

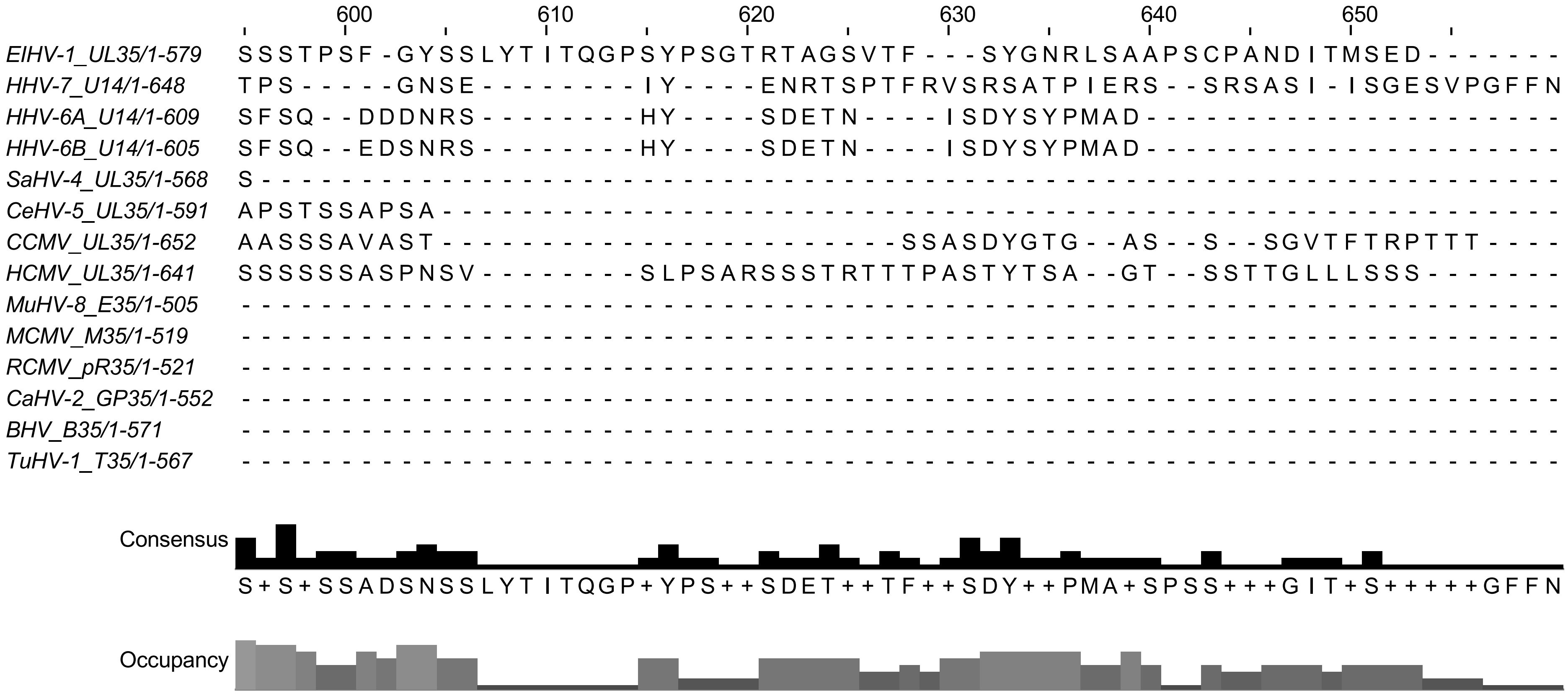
600 610 620 630 640 650
ElHV-1_UL35/1-579 SSSTPSF-GYSSLYTITQGPSYPSGTRTAGSVTF---SYGNRLSAAPSCPANDITMSED-------
HHV-7_U14/1-648 TPS-----GNSE---------IY----ENRTSPTFRVSRSATPIERS--SRSASI-ISGESVPGFFN
HHV-6A_U14/1-609 SFSQ--DDDNRS--------HY----SDETN----ISDYSYPMAD--------------------
HHV-6B_U14/1-605 SFSQ--EDSNRS--------HY----SDETN----ISDYSYPMAD--------------------
SaHV-4_UL35/1-568 S--
CeHV-5_UL35/1-591 APSTSSAPSA---
CCMV_UL35/1-652 AASSSAVAST-------------------SSASDYGTG--AS--S--SGVTFTRPTTT----
HCMV_UL35/1-641 SSSSSSASPNSV--------SLPSARSSSTRTTTPASTYTSA--GT--SSTTGLLLSSS-------
MuHV-8_E35/1-505 ---
MCMV_M35/1-519 ---
RCMV_pR35/1-521 ---
CaHV-2_GP35/1-552 ---
BHV_B35/1-571 ---
TuHV-1_T35/1-567 ---
Consensus
S+S+SSADSNSSLYTITQGP+YPS++SDET++TF++SDY++PMA+SPSS+++GIT+S+++++GFFN
Occupancy

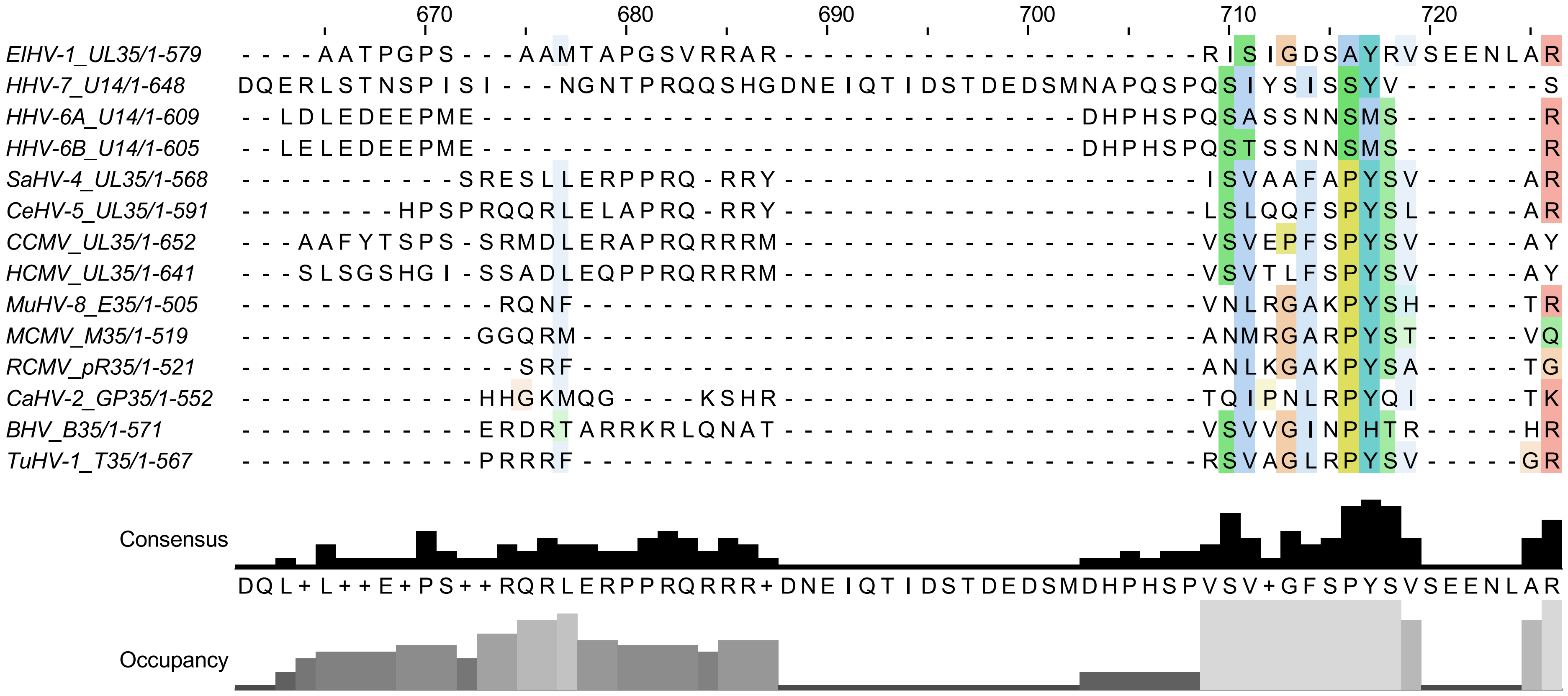

670
680
690
700
710
720
EIHV-1_UL35/1-579 ----AATPGPS---AAMTAPGSVRRAR--------------------RISIGDSAYRVSEENLAR
HHV-7_U14/1-648 DQERLSTNSPISI---NGNTPRQQSHGDNEIQTIDSTDEDSMNAPQSPQSIYSISSYV-------S
HHV-6A_U14/1-609 --LDLEDEEPME------------------------------DHPHSPQSASSNNSMS-------R
HHV-6B_U14/1-605 --LELEDEEPME------------------------------DHPHSPQSTSSNNSMS-------R
SaHV-4_UL35/1-568 -----------SRESLLERPPRQ-RRY--------------------ISVAAFAPYSV-----AR
CeHV-5_UL35/1-591 --------HPSPRQQRLELAPRQ-RRY--------------------LSLQQFSPYSL-----AR
CCMV_UL35/1-652 ---AAFYTSPS-SRMDLERAPRQRRRM--------------------VSVEPFSPYSV-----AY
HCMV_UL35/1-641 ---SLSGSHGI-SSADLEQPPRQRRRM--------------------VSVTLFSPYSV-----AY
MuHV-8_E35/1-505 -------------RQNF------------------------------VNLRGAKPYSH-----TR
MCMV_M35/1-519 ------------GGQRM------------------------------ANMRGARPYST-----VQ
RCMV_pR35/1-521 --------------SRF------------------------------ANLKGAKPYSA-----TG
CaHV-2_GP35/1-552 ------------HHGKMQG----KSHR--------------------TQIPNLRPYQI-----TK
BHV_B35/1-571 ------------ERDRTARRKRLQNAT--------------------VSVVGINPHTR-----HR
TuHV-1_T35/1-567 ------------PRRRF------------------------------RSVAGLRPYSV-----GR
Consensus
DQL+L++E+PS++RQRLERPPRQRRR+DNEIQTIDSTDEDSMDHPHSPVSV+GFSPYSVSEENLAR
Occupancy

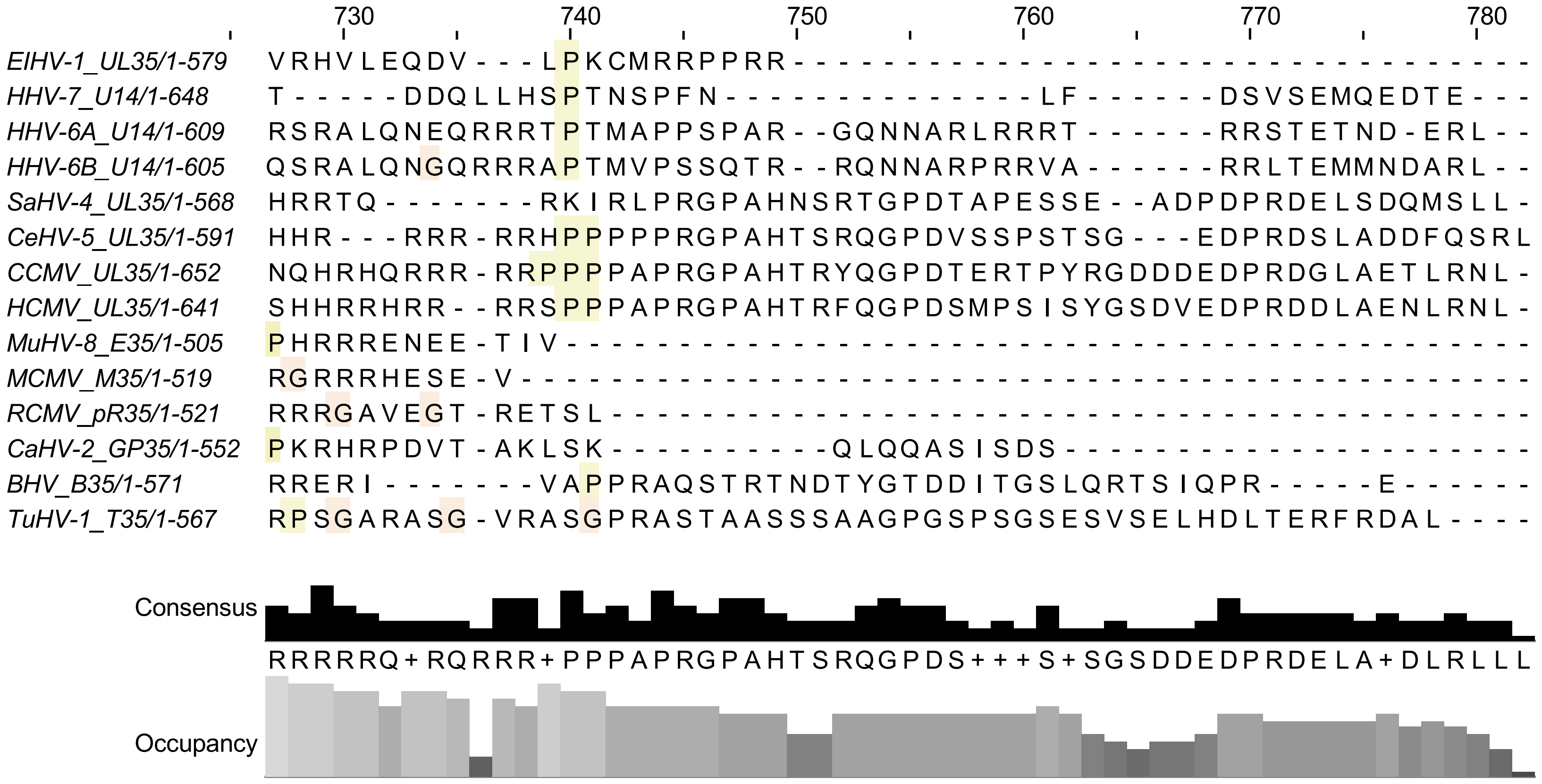

730 740 750 760 770 780
ElHV-1_UL35/1-579 VRHVLEQDV---LPKCMRRPPRR-----------------------------------
HHV-7_U14/1-648 T-----DDQLLHSPTNSPFN---------------LF------DSVSEMQEDTE---
HHV-6A_U14/1-609 RSRALQNEQRRRTPTMAPPSPAR--GQNNARLRRRT------RRSTETND-ERL--
HHV-6B_U14/1-605 QSRALQNGQRRRAPTMVPSSQTR--RQNNARPRRVA------RRLTEMMNDARL--
SaHV-4_UL35/1-568 HRRTQ-------RKIRLPRGPAHNSRTGPDTAPESSE--ADPDPRDELSDQMSLL-
CeHV-5_UL35/1-591 HHR---RRR-RRHPPPPPRGPAHTSRQGPDVSSPSTSG---EDPRDSLADDFQSRL
CCMV_UL35/1-652 NQHRHQRRR-RRPPPPAPRGPAHTRYQGPDTERTPYRGDDDEDPRDGLAETLRNL-
HCMV_UL35/1-641 SHHRRHRR--RRSPPPAPRGPAHTRFQGPDSMPSISYGSDVEDPRDDLAENLRNL-
MuHV-8_E35/1-505 PHRRRENEE-TIV---
MCMV_M35/1-519 RGRRRHESE-V---
RCMV_pR35/1-521 RRRGAVEGT-RETSL---
CaHV-2_GP35/1-552 PKRHRPDVT-AKLSK----------QLQQASISDS---------------------
BHV_B35/1-571 RRERI-------VAPPRAQSTRTNDTYGTDDITGSLQRTSIQPR-----E------
TuHV-1_T35/1-567 RPSGARASG-VRASGPRASTAASSSAAGPGSPSGSESVSELHDLTERFRDAL----
Consensus
RRRRRQ+RQRRR+PPPAPRGPAHTSRQGPDS+++S+SGSDDEDPRDELA+DLRLLL
Occupancy
